# The epsomitic phototrophic microbial mat of Hot Lake, Washington: community structural responses to seasonal cycling

**DOI:** 10.3389/fmicb.2013.00323

**Published:** 2013-11-13

**Authors:** Stephen R. Lindemann, James J. Moran, James C. Stegen, Ryan S. Renslow, Janine R. Hutchison, Jessica K. Cole, Alice C. Dohnalkova, Julien Tremblay, Kanwar Singh, Stephanie A. Malfatti, Feng Chen, Susannah G. Tringe, Haluk Beyenal, James K. Fredrickson

**Affiliations:** ^1^Biological Sciences Division, Fundamental and Computational Sciences Directorate, Pacific Northwest National LaboratoryRichland, WA, USA; ^2^Chemical, Biological, and Physical Sciences Division, National Security Directorate, Pacific Northwest National LaboratoryRichland, WA, USA; ^3^Scientific Resources Division, William R. Wiley Environmental Molecular Sciences Laboratory, Pacific Northwest National LaboratoryRichland, WA, USA; ^4^Lawrence Berkelely National Laboratory, Joint Genome InstituteWalnut Creek, CA, USA; ^5^The Gene and Linda Voiland School of Chemical Engineering and Bioengineering, Washington State UniversityPullman, WA, USA

**Keywords:** Hot Lake, phototrophic microbial mats, 16S tag sequencing, phylogenetic turnover, microbial diversity, seasonal cycling, community assembly, magnesium sulfate

## Abstract

Phototrophic microbial mats are compact ecosystems composed of highly interactive organisms in which energy and element cycling take place over millimeter-to-centimeter-scale distances. Although microbial mats are common in hypersaline environments, they have not been extensively characterized in systems dominated by divalent ions. Hot Lake is a meromictic, epsomitic lake that occupies a small, endorheic basin in north-central Washington. The lake harbors a benthic, phototrophic mat that assembles each spring, disassembles each fall, and is subject to greater than tenfold variation in salinity (primarily Mg^2+^ and SO^2−^_4_) and irradiation over the annual cycle. We examined spatiotemporal variation in the mat community at five time points throughout the annual cycle with respect to prevailing physicochemical parameters by amplicon sequencing of the V4 region of the 16S rRNA gene coupled to near-full-length 16S RNA clone sequences. The composition of these microbial communities was relatively stable over the seasonal cycle and included dominant populations of *Cyanobacteria*, primarily a group IV cyanobacterium (*Leptolyngbya*), and *Alphaproteobacteria* (specifically, members of *Rhodobacteraceae* and *Geminicoccus*). Members of *Gammaproteobacteria* (e.g., *Thioalkalivibrio* and *Halochromatium*) and *Deltaproteobacteria* (e.g., *Desulfofustis*) that are likely to be involved in sulfur cycling peaked in summer and declined significantly by mid-fall, mirroring larger trends in mat community richness and evenness. Phylogenetic turnover analysis of abundant phylotypes employing environmental metadata suggests that seasonal shifts in light variability exert a dominant influence on the composition of Hot Lake microbial mat communities. The seasonal development and organization of these structured microbial mats provide opportunities for analysis of the temporal and physical dynamics that feed back to community function.

## Introduction

Microbial mats are macroscale communities of metabolically linked organisms (Taffs et al., 2009; Klatt et al., 2013) occupying a shared biogenic ultrastructure typically composed of an organic exopolymeric matrix (Decho et al., 2005; Braissant et al., 2009). As such, microbial mats exist as entire ecosystems where complete energy and element cycles, otherwise taking place over large distances, occur on millimeter scales (reviewed in Franks and Stolz, 2009; Paerl and Yannarell, 2010). Consequently, the diverse metabolic activities of the community members impose steep physical and chemical gradients and create niches with fine spatiotemporal resolution (Dupraz et al., 2009). Sunlight drives strong vertical community structuring of phototrophic microbial mats as photons of specific wavelengths are selectively harvested with depth (e.g., Pierson et al., 1987; Jorgensen and Des Marais, 1988). These mats experience significant variation in their physicochemical environments and, therefore, the interspecies interactions operating within them, as light availability changes over diel and seasonal cycles (Van der Meer et al., 2005; Villanueva et al., 2007; Steunou et al., 2008; Dillon et al., 2009). Cyanobacteria often populate the upper, photic areas in these mats where they capture solar energy, fixing carbon and producing O_2_ as a byproduct; both products of photosynthesis are subsequently cycled by heterotrophs (Paerl et al., 2000). Cyanobacterial mats are common in hypersaline systems worldwide, where elevated salinities restrict grazers and allow accretion of biomass (Oren, 2010).

The biology of mat communities occupying athalassohaline environments, especially those dominated by Mg-Na-SO_4_ brines, remains understudied considering their widespread global occurrence. Epsomitic hypersaline lakes and playas are common features of the inter-range semi-arid plateau between the Rocky Mountains and the Pacific Coast and Cascade Ranges that stretches from eastern Washington and Oregon through British Columbia (Bauld, 1981; Renaut, 1990) and within the endorheic Ebro Basin in Spain (Guerrero and De Wit, 1992; Jonkers et al., 2003). Insofar as athalassohaline mat systems in western North America have been studied in detail, the focus has predominantly been on carbonate precipitation (Renaut, 1993; Power et al., 2007, 2009) or the detection of signatures of life from an astrobiological aspect (Foster et al., 2010).

Hot Lake is a heliothermal hypersaline lake in extreme north-central Washington near Oroville that seasonally harbors a benthic phototrophic microbial mat. It is constrained within a glacially-carved, endorheic basin in a semi-arid climatic zone. The basin drains less than a one-half square mile (<1.3 km^2^) watershed and is underlain by metamorphic rock, dolomites, and shales (Jenkins, 1918). Sulfuric acid produced by the oxidation of neighboring pyrite and pyrrhotite deposits releases magnesium, calcium, and sulfate ions that are transported into the lake (Jenkins, 1918). Due to the endorheic nature of the basin, these salts accumulate in Hot Lake, causing it to become magnesium-dominated as calcium sulfate precipitates. Positive net evaporation through the summer and early fall months causes significant decreases in water volume and concurrent increases in salinity (Anderson, 1958). Hot Lake is meromictic with a relatively fresh mixolimnion situated atop a more saline monimolimnion (Anderson, 1958; Walker, 1974). The lake exhibits an inverse thermal gradient, sometimes exceeding 50°C at maximum, due to peak light absorption in the upper monimolimnion and insulation by the overlying water column (Anderson, 1958). Retention of heat in the monimolimnion likewise causes some of the warmest solar-heated waters on Earth at Solar Lake (Cohen et al., 1977), which is also home to a well-studied benthic cyanobacterial mat that exhibits seasonal cycling (Krumbein et al., 1977; Jorgensen et al., 1979, 1986; Frund and Cohen, 1992).

Hot Lake has previously been studied in some detail for its unique limnology and geology (Handy, 1916; Jenkins, 1918; Anderson, 1958; Bennett, 1962; Walker, 1974), as well as for the flora (St. John and Courtney, 1924; McKay, 1935; Anderson, 1958) and fauna (Anderson, 1958; Broch, 1969) that inhabit the lake. Recent work also includes the microbes of its marginal soils (Kilmer et al., in press). To date, however, only Anderson mentioned Hot Lake's benthic microbial mat; his study identified the mat's cyanobacteria but did not characterize the non-cyanobacterial microbial populations inhabiting the mat. In 1955, the mat was present at depths exceeding 1.0 m and extended into the upper reaches of the monimolimnion (Anderson, 1958, 2012). In this work, we interrogate the mat community's compositional variation as it responds to the highly dynamic environmental conditions of Hot Lake throughout the seasonal cycle of 2011. Additionally, we examine the community's phylogenetic turnover with respect to the environmental metadata and infer processes driving community variation.

## Materials and methods

### Sampling and environmental characterization

**Figure 1:**
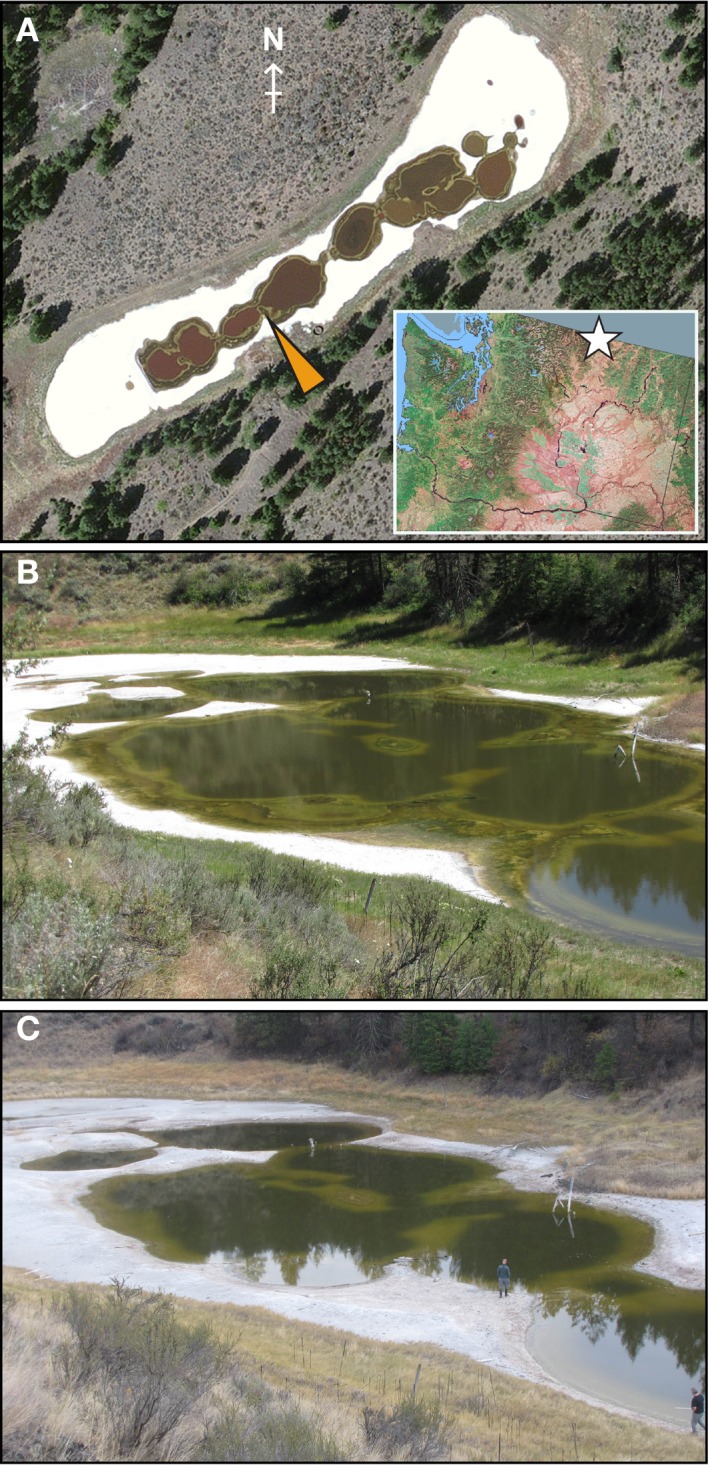
**Physical characteristics of Hot Lake. (A)** Aerial photograph of Hot Lake on August 6, 2011 showing the surrounding mixed grass and pine communities common within its endorheic basin and the gypsum flats flanking the lake. Mat was sampled at the location indicated by the yellow arrowhead. On the inset map of the state of Washington, the location of Hot Lake is represented by a white star. QuickBird imagery was provided by DigtalGlobe and Land Info Worldwide Mapping, inset map from the National Atlas of the United States. Seasonal changes in water level can be clearly seen from photographs of the north-easternmost basin of Hot Lake taken on July 7, 2011 **(B)** and October 20, 2011 **(C)**.

Benthic mat samples were collected on April 21, July 7, September 1, and October 20, 2011 at the same sampling station, located at 48.973062°N, 119.476876°W at an elevation of ~576 m. Mixolimnion water and ice were also collected from the same location on December 1, 2011. The collected mat was visually representative of mat observed ringing the entire lake (See Figure 1A). The mat was operationally defined as the portion that remained intact when lifted off the underlying sediments and was typically 3–5 mm in thickness. Two samples of mat (~50 cm^2^ each) were collected per time point, cryoprotected by immersion in 2.3 M sucrose, and immediately frozen on dry ice. Mat collected for microscopic analysis was fixed in the field with 4% paraformaldehyde in lake water and held at 4°C for at least 24 h to ensure complete fixation. Paired 50-mL water samples were taken from the same depth as sampled mat. The water temperature was immediately recorded using a WTW 3400i Multi-Parameter Field Meter (WTW, Inc., College Station, TX) prior to storing the samples at 4°C. Samples were then filtered in the laboratory using a 0.22-μm polyethersulfone syringe filter unit (EMD Millipore, Billerica, MA) and held at 4°C. Filtrate was assayed for pH, total dissolved solids (TDS), alkalinity, major cations (magnesium, sodium, potassium, calcium), major anions (sulfate, chloride), dissolved organic carbon (DOC), nitrate, ammonium, and o-phosphate by Huffman Laboratories (Golden, CO). Irradiance data were obtained from OVLW1, a remote automated weather station ~1.5 km from Hot Lake at an elevation of ~440 m. These data were provided by the U. S. Bureau of Land Management & Boise Interagency Fire Center and hosted by MesoWest, a project of the Department of Atmospheric Sciences at the University of Utah (http://mesowest.utah.edu).

### Fiber optic microprofiling

A custom-built fiber optic microprobe was used to quantify light penetration by depth in the mat. The fiber optic microprobes, which had tapered tips, were formed using a variation on previously described techniques (Gao et al., 1995; Beyenal et al., 2000), as detailed in Lewandowski and Beyenal (2007). Briefly, the insulation near the tip of a 9 μm core fiber with numerical aperture (NA) of 0.11 (Corning^®^ SMF-28^®^ ULL optical fiber, Corning, NY, USA) was mechanically stripped, the tip cleaned with isopropyl alcohol and cleaved. The cleaved fiber was held vertically in a precision linear positioner and lowered into unstirred, 37.5% hydrofluoric acid. After etching for 2–15 min at room temperature, the fiber was removed and rinsed in deionized water. Ambient light intensity was then measured; stable, reproducible readings and inspection using a scanning electron microscope indicated the successful formation of the fiber tip. The optical fiber cable was connected using an FC connector to an Ocean Optics Torus Miniature Spectrometer (Dunedin, FL, USA). The fiber optic microprobe was placed on a micromanipulator controlled by a stepper motor controller (PI M-230.10S Part No. M23010SX, Physik Instrumente, Auburn, MA, USA) and custom Microprofiler^®^ software. Spectra were taken at 0.25-mm increments throughout the mat. Light intensity directly above the mat surface was recorded as a reference. Intensity values at different depths are reported as percent transmission relative to the surface illumination for each wavelength.

### Cryosectioning

To prepare cross sections for microscopic analysis, paraformaldehyde-fixed mat was cryoprotected overnight with 2.3 M sucrose at 4°C. Blocks of cryoprotected mat were excised from the center of the mat sample and embedded in Tissue-Tek O.C.T. Compound in Tissue-Tek 10 × 10 × 5-mm Cryomolds (Electron Microscopy Sciences, Hatfield, PA). 50-μm-thick sections were cut using a Leica CM1520 cryostat, transferred to slides, mounted in VECTASHIELD mounting medium (Vector Laboratories, Burlingame, CA) and imaged using a Nikon Optiphot-2 epifluorescence microscope. Depth-resolved sections for DNA extraction were prepared by excising blocks from the center of frozen, cryoprotected mat samples and embedding them in O.C.T. Compound in 25 × 20 × 5-mm Tissue-Tek Cryomolds (Electron Microscopy Sciences, Hatfield, PA) oriented with the pinnacled top facing up. The mat was then sectioned into 50-μm-thick sections, 10 of which were pooled to span 500 μm total depth, and nucleic acids were extracted as described below.

### Subsampling and dna extraction

Seasonal cycling of the mat community was examined by extracting genomic DNA from cryoprotected samples of whole mat. Frozen, cryoprotected mat was subsampled into 3 × 3 grids, each of the nine subsamples being 0.5 mm on a side. For each time point, three sections of each grid were randomly chosen from each of two plates using a random number generator (www.random.org, see Figure 5A). DNA was extracted according to the enzymatic protocol (EP) previously described (Ferrera et al., 2010) with the following modifications: prior to purification, ~100-mg mat samples were washed with molecular biology grade 0.5 M EDTA at pH 8.0 (Life Technologies, Carlsbad, CA) to remove excess magnesium and resuspended in lysis buffer (50 mM Tris at pH 8.0, 25 mM EDTA pH 8.0). Samples were then incubated at 85°C for 5 min to inactivate native nucleases and slowly cooled to 37°C. Chemical and enzymatic lysis then proceeded as described by Ferrera et al. Briefly, samples were treated with 1 mg/ml lysozyme at 37°C for 45 min, at which point 1:10 vol 10% SDS and 0.2 mg/ml proteinase K were added prior to incubation at 56°C for 1 h. Post-lysis, DNA was extracted with phenol-chloroform-isoamyl alcohol (25:24:1, vol:vol:vol) and then chloroform-isoamyl alcohol (24:1). Sodium acetate at pH 5.5 was added to a final concentration of 0.3 M. The DNA was then precipitated in 50% isopropanol, washed in 70% ethanol, dried, and resuspended in TE buffer (10 mM Tris-HCl at pH 8.0, 1 mM EDTA). DNA was extracted from cryosectioned laminar sections using the same protocol with the exception that, prior to EDTA washing, samples were washed three times with 50 mM Tris at pH 8.0 in 25% sucrose to remove the O.C.T. Compound.

### Clone library construction, sequencing, and processing

Near-full-length *rrnA* genes were PCR amplified from genomic DNA harvested from a ~25-mm^2^ (238 mg) whole-mat sample collected on July 7, 2011 using universal bacterial primers 27F (5′-AGAGTTTGATCMTGGCTCAG-3′) and 1492R (5′-GGYTACCTTGTTACGACTT-3′) (Lane, 1991). PCR was performed using Phusion polymerase (New England Biolabs, Ipswitch, MA) in HF Buffer and 3% dimethyl sulfoxide according to the manufacturer's instructions at an annealing temperature of 55°C for 27 cycles. PCR product was cloned using the Zero Blunt TOPO PCR cloning kit (Life Technologies, Carlsbad, CA) according to the manufacturer's directions. Plasmids were isolated from clones and their 16S rRNA genes were sequenced using Sanger dideoxy chain-termination sequencing by Functional Biosciences (Madison, WI) from pCR-II-TOPO's SP6 and T7 promoter regions. Using the ContigExpress algorithm of Vector NTi Advance v. 11.0 (Life Technologies, Carlsbad, CA), sequence ends were trimmed until the initial and final 25 bases contained no ambiguities or bases with a Phred quality score of less than 20. Sequences were then checked for vector contamination and assembled into contigs. Assemblies were curated and mismatches resolved manually.

Post assembly, sequences were aligned using the mothur-formatted SILVA-based bacterial reference alignment (http://www.mothur.org/w/images/9/98/Silva.bacteria.zip, updated April 22, 2012) in mothur v. 1.29 (Schloss et al., 2009). These aligned sequences were filtered to remove non-informative columns and clustered to account for the expected error for a Phred score of 20 (1%, allowing 12 differences across the alignment). Sequences were then checked for chimeras using UCHIME (Edgar et al., 2011) as implemented in mothur 1.29 both in self-referential mode and using the SILVA gold alignment as a reference. Chimeras detected using the reference sequences were manually examined to prevent the inadvertent removal of sequences without good reference sequences. Near-full-length clones that were observed at least twice in the clone library (at >99% identity) or that mapped >0.1% of the Itag sequences were chosen for more thorough analysis, and these were manually examined for chimeras prior to submission to GenBank (see Supplemental Table 1 for accession numbers). The full-length, 50,000-column alignment of these representative sequences was incorporated into the reference alignment used in the Itag analysis in order to promote the alignment of Itag sequences similar to these near-full-length sequences. In addition, it was degapped and used as a reference to map Itag sequences (see following section).

### Itag sequencing

Short 16S rRNA tag (Itags) sequencing was done on an Illumina MiSeq instrument at the Joint Genome Institute, Walnut Creek, CA. Primer design for universal amplification of the V4 region of 16S rDNA was based on a protocol published by Caporaso and co-workers (Caporaso et al., 2011). The forward primer (515F, 5′- AATGATACGGCGACCACCGAGATCTACAC TATGGTAATT GT GTGCCAGCMGCCGCGGTAA) remained unchanged and the barcoded reverse primers are largely similar to the Caporaso V4 reverse primer (806R), but with 0–3 random bases and the Illumina sequencing primer binding site added between the amplification primer and the Illumina adapter sequence. For each sample, three separate 16S rRNA amplification reactions targeting the V4 hypervariable region were performed, pooled together, cleaned up using AMPureXP (Beckman Coulter) magnetic beads, and quantified with the Qubit HS assay (Invitrogen). Some samples were also analyzed with a BioAnalyzer 2100 (Agilent) instrument to confirm appropriate amplicon size. Pooled amplicons were then diluted to 10 nM and quantified by qPCR prior to sequencing according to JGI's standard procedures. A total of 3,184,278 (1,592,139 forward and 1,592,139 reverse reads) barcoded paired-end reads where obtained after computational removal of PhiX and contaminant reads (reads containing Illumina adapters). Reads were then paired-end assembled using FLASH (Magoc and Salzberg, 2011). All sequences were then trimmed from both 5′ and 3′ ends using a sliding window of 10 bp and quality score threshold of 33. Reads having more than 5 ambiguous bases, an average quality score lower than 30, or more than 10 nucleotides having a quality score lower than 15 were rejected. We ended with a total of 1,634,356 quality-filtered tag sequences that were used for downstream analyses.

### Itag sequence processing and analysis

Sequences were processed using mothur v. 1.29 as previously described (Schloss et al., 2011), though some modifications were made to accommodate 2 × 250 cycle paired-end MiSeq sequences, and 454-specific portions of the protocol were omitted. Paired, phiX-decontaminated reads were sorted into samples by barcode using a custom Perl script and mothur-formatted FASTA and group files were generated. The FASTA file was filtered to remove those sequences with ambiguities or those with lengths shorter than 251 nts. Thereafter, processing closely followed the protocol of Schloss et al. (2011), with the exception that sequences were subsampled prior to distance matrix generation. Briefly, sequences were aligned to the SILVA-based bacterial reference alignment, which was augmented with the Hot Lake mat near-full-length sequences (see Clone library construction, sequencing, and processing above). Sequences were then screened to remove those that did not align to positions 13871–23444 of the reference alignment, filtered to remove non-informative columns, pre-clustered to >99% identity (allowing two differences), and dereplicated. Sequences were then checked for chimeras using UCHIME as implemented in mothur 1.29 in self-referential mode and identified chimeras were removed.

The resulting set of filtered sequences was then classified using a Wang (Bayesian) approach with the Ribosome Database Project training set v. 9 (updated March 20, 2012 and formatted for mothur) as a reference. Sequences of unknown classification at the kingdom level were removed. Each group was then subsampled to the size of the smallest group (14,562 sequences). Sequences were clustered into operational taxonomic units (OTUs) using an average neighbor algorithm with a 3% cutoff classified at a cutoff bootstrap value of 80%. Alpha (species observed, inverse Simpson, and Simpson evenness) and beta diversity metrics (Bray-Curtis) were computed in mothur using subsampled sequences (*n* = 14.562). Twenty clones from the Hot Lake mat clone library were selected based upon abundance in the library and representation of phyla and evenly pooled to generate a mock community. The mock community was amplified by PCR as described above and sequenced alongside the other Itag samples to compute the sequencing error rate.

### Short read mapping and phylogeny reconstruction

Unique Itag reads were mapped to the near-full-length *rrnA* sequences from the Hot Lake mat clone library using the nucmer algorithm in MUMmer v. 3.23 (Kurtz et al., 2004). A match was defined as a minimum identity of 99% across at least 243 nts. These sequences and their corresponding counts were compared with their OTU assignments, and the percent of the reads composing each OTU that mapped to each sequence from the clone library was calculated. Near-full-length sequences that were mapped by >1% of the reads of the most abundant OTUs and their near neighbors were aligned. A phylogeny was then reconstructed using a neighbor-joining algorithm assuming a maximum composite likelihood substitution model, including transitions and transversions at uniform rates among sites and pairwise deletion of gaps, within MEGA5.1 (Tamura et al., 2011). MEGA5.1 was also used to reconstruct a maximum likelihood phylogeny using the nearest-neighbor interchange heuristic and general time reversible (GTR) substitution model assuming uniform substitution rates at all sites. The robustness of both phylogenies was tested using the bootstrap method with 1000 replications.

### Phylogenetic null model analysis

The OTU table was rarefied and representative sequences for each of the 993 most abundant OTUs were retrieved from the Itags, placed within a maximum-likelihood phylogeny using FastTree 2.1 (Price et al., 2010), and used to quantify Bray and Curtis (1957) dissimilarity for all between-community pairwise comparisons. Mantel tests were used to relate Bray-Curtis values to between-community environmental differences in order to evaluate the degree to which community composition varied with environmental conditions. Bray-Curtis values were related to each environmental variable independently, and significance was evaluated with a permutation-based test to control for data non-independence.

A non-significant relationship between Bray-Curtis and a given environmental variable suggests that the environmental variable being evaluated does not strongly influence community composition. All environmental variables were, however, significantly related to Bray-Curtis (see Results). Bray-Curtis analyses therefore, provided relatively little information regarding the identity of environmental variables that most strongly influence community composition. To gain more insight, we coupled turnover in the phylogenetic structure of communities with a randomization approach that provides an expected magnitude of phylogenetic turnover when community composition is governed primarily by stochastic factors (for conceptual and technical details see Stegen et al., 2012, 2013; Swenson et al., 2012).

Phylogenetic turnover was quantified as the abundance-weighted-mean phylogenetic distance among closest relatives occurring in two communities, the β-Mean Nearest Taxon Distance (βMNTD) (for details see Fine and Kembel, 2011; Webb et al., 2011; Stegen et al., 2012). To derive ecological information from phylogenetic turnover, we compared observed βMNTD to expected βMNTD under a model of stochastic community assembly. A distribution of expected values was found using 999 iterations of a randomization that moved OTU names across tips of the phylogeny.

The β-Nearest Taxon Index (βNTI) quantifies the difference between observed and expected βMNTD in units of standard deviations; negative and positive βNTI values indicate less than and greater than expected phylogenetic turnover, respectively. Stochastic aspects of community assembly are controlled for in the randomization, such that a significant increase in βNTI over increasing environmental differences provides good evidence that variation in environmental conditions causes alterations in community composition by selecting for particular OTUs (Stegen et al., 2012). To complement the Bray-Curtis analyses, we therefore, used Mantel tests to relate βNTI to environmental variables one at a time, and permutations were used to evaluate significance.

It is important to note that the use of βNTI to arrive at ecological inferences makes the assumption that phylogenetic relationships carry ecological information. This assumption was tested using a phylogenetic Mantel correlogram (as in Stegen et al., 2013; Wang et al., 2013), which relates among-OTU ecological differences to among-OTU phylogenetic distances. OTU ecological distances were quantified as in Stegen et al. (2013) and Wang et al. (2013) using all measured environmental variables. When OTU ecological differences are significantly related to between-OTU phylogenetic distances, there is said to be “phylogenetic signal” (Losos, 2008).

## Results

### Description of hot lake and its phototrophic mat

**Figure 2:**
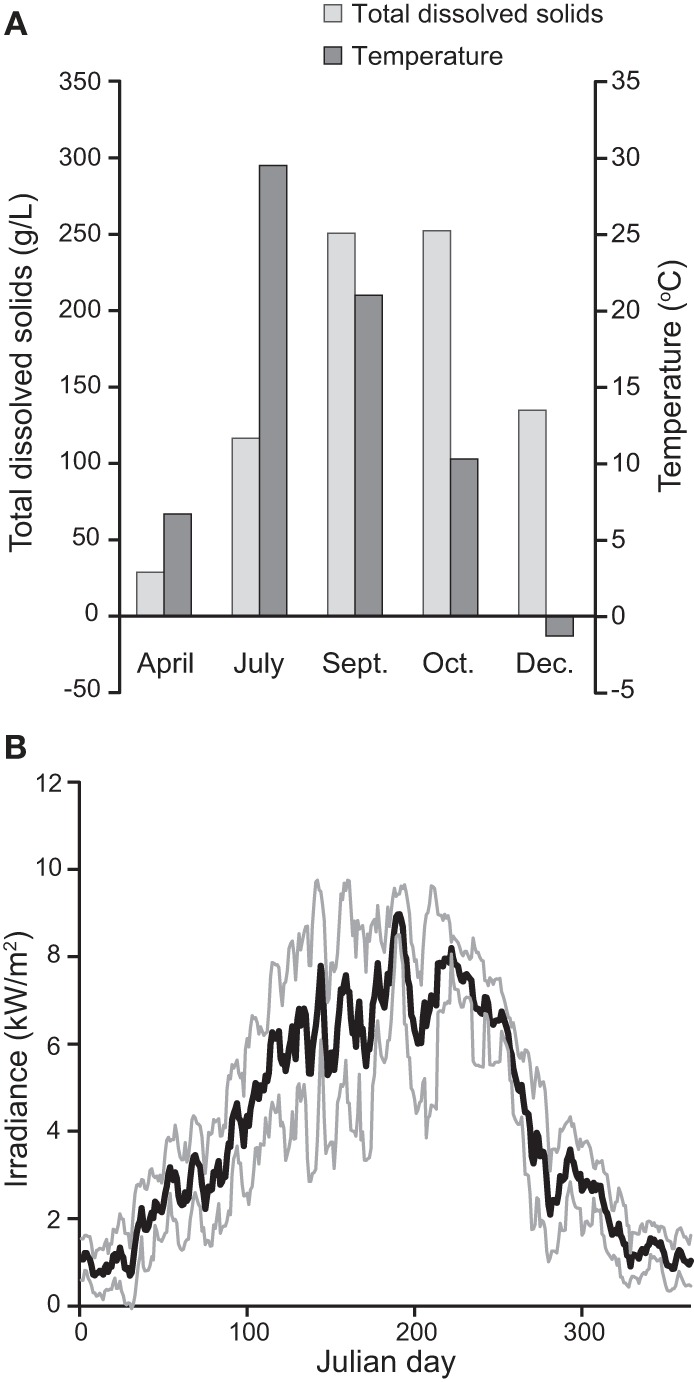
**Seasonal variation in the environmental conditions experienced by the mat community in Hot Lake. (A)** Variation in salinity (as represented by total dissolved solids) and temperature in water proximal to sampled mat. December values are from water immediately below ice cover. **(B)** Variation in irradiance throughout 2011 as recorded by remote automated weather station OVLW1. Maximal recorded daily irradiance near Hot Lake was 9574 W/m^2^ on June 26, while just 160 W/m^2^ was recorded at minimum on January 7.

We observed very different water levels at Hot Lake than those described by Anderson. Most notably, the maximum water level in 2011 was ~1 m lower than in 1955 (Anderson, 1958); surfaces that Anderson reported as submerged <1 m deep we found to be exposed and covered by fine white crystals (Figure 1A). Periodic descriptions of Hot Lake by others over the course of a half-century (St. John and Courtney, 1924; McKay, 1935; Walker, 1974), coupled with aerial photography, suggest that Hot Lake's water level in 2011 was more typical of modern trends. As the first half of the 1950s exhibited colder-than-average temperatures and elevated levels of precipitation in the region (Anderson, 2012), it is likely that Anderson observed Hot Lake near the upper bounds of its water volume. The white efflorescent salts on the surface of the lake's dehydrated banks, which others have previously described (Jenkins, 1918; St. John and Courtney, 1924; McKay, 1935), we determined to be primarily composed of gypsum, epsomite, hexahydrite, aragonite, and magnesite by X-ray diffraction analysis (data not shown). The salinity of Hot Lake (reported as TDS) of water collected at equal depths with the sampled mat was at its seasonal minimum in spring after significant inflow from precipitation and snowmelt (Figure 2A, Table 1). Salinity increased throughout 2011, driven by escalating evaporation and decreasing water levels (Figures 1B,C) over the summer and into fall. Day-to-day variability in irradiance was most strongly affected by cloud cover, which was less influential during late summer and fall than earlier in the year (Figure 2B). Mat-level water temperature was closely associated with irradiance (cf. Figure 2 and Anderson, 1958). The concentrations of major cations (Mg^2+^, Na^+^, K^+^), anions (SO^2−^_4_, Cl^−^), and alkalinity all correlated to TDS and displayed strong evidence of evaporitic concentration throughout the seasonal cycle (Table 1). The DOC in Hot Lake also showed evidence of evaporative concentration, reaching 23.5 mM in mixolimnion water in September of 2011, which indicates the system was unlikely to be carbon-limited. In contrast, dissolved nitrogen sources (i.e., NO^−^_3_ and NH^+^_4_) and o-phosphate concentrations were very near or below the detection limits, suggesting either may be limiting for mat growth. In the case of phosphate, this effect is likely imposed by the sparing solubility of magnesium and calcium phosphates.

**Table 1:** **Aqueous geochemical analysis of mat-level waters**.

			**Sample collection date**					
			**4 April**	**7 July**	**1 September**	**20 October**	**1 December**	**1 December^a^**
General water chemistry	pH	units	8.37	8.52	8.15	8.10	8.37	8.65
	TDS^b^	(g/L)	28.8	116.6	250.6	252.1	134.7	53.1
	Alkalinity	(mM)	3.00	9.19	18.08	18.78	9.16	3.80
Major cations	Mg^2+^	(mM)	158	671	1453	1444	728	295
	Na^+^	(mM)	81	318	713	718	364	146
	K^+^	(mM)	12	43	103	102	49	20
	Ca^2+^	(mM)	9	16	13	13	10	4
Major anions	SO^2−^_4_	(mM)	198	756	1758	1796	884	356
	Cl^−^	(mM)	19	57	115	116	66	28
Dissolved carbon	DOC^c^	(mM C)	3.2	12.7	23.5	23.4	11.5	4.8
	Bicarbonate	(mM)	5.6	10.0	11.5	14.6	9.1	3.9
	Carbonate	(mM)	<0.8	4.2	12.3	11.5	4.6	1.8
Nutrients	Nitrate	(mM)	<0.36	<0.36	<0.36	<0.36	<0.36	<0.36
	Ammonia	(mM)	0.04	0.03	0.03	0.02	0.04	0.04
	o-phosphate	(mM)	<0.52	<0.52	<0.52	<0.52	<0.52	<0.52

Samples collected over a seasonal cycle highlight the large differences in concentration of dominant cations, anions, and dissolved carbon species. While organic and inorganic carbon concentrations remain high throughout the sampling period, nutrient levels (including nitrate, ammonia, and o-phosphate) are persistently low and may limit microbial growth. The strong correlation between TDS and most major cations and anions are consistent with evaporitic concentration. Charge balance was checked and was within 5% at every sampling point.

a Melted ice from lake surface.

b Total dissolved solids.

c Dissolved organic carbon.

The distribution of the microbial mat relative to depth varied over the seasonal cycle. As the year progressed, the mat gradually colonized increasingly shallower benthic surfaces, beginning near the thermocline and proceeding upward toward the water line. In April, these sediments were free of mat and consisted of a thin layer of gypsum and carbonate. At that time, the mat was present at a minimum depth of 60 cm. By early July, it had colonized approximately the lower half of the benthic surfaces in contact with the mixolimnion and proceeded to occupy all submerged sediments above the thermocline by September. The declining water level left shallow mat exposed by late summer; desiccated mat was widespread in October. Though ice covered the lake in December, frozen mat was present in the ice and immediately below it. Once ice cover receded the following April (2012), we again found no evidence of a benthic mat in the oxic mixolimnion. As the water level varied significantly throughout 2011, we used fixed points of reference to correlate mat coverage to absolute position on the lake bottom. We observed this mat community assembly-disassembly cycle again from April, 2012 to April, 2013.

**Figure 3:**
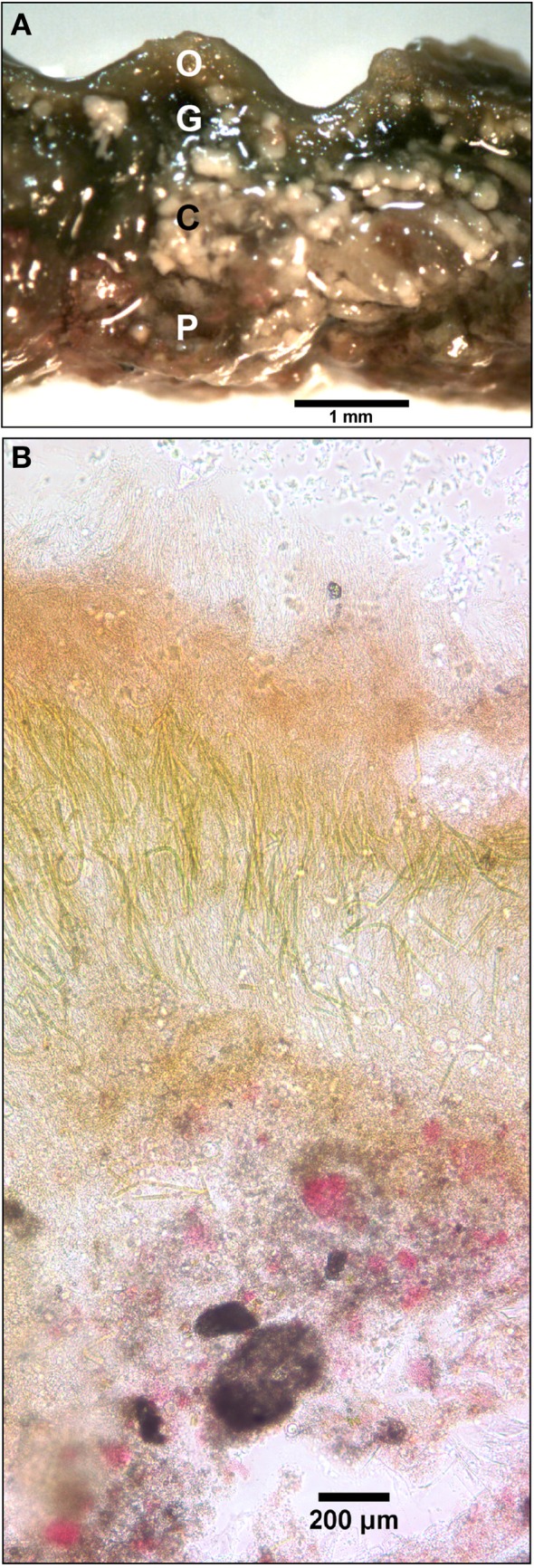
**Ultrastructure of the Hot Lake mat sampled on September 1, 2011. (A)** Cross-section of the Hot Lake mat at the millimeter scale. Orange (O), green (G), and pink (P) lamina are readily apparent along with interspersed carbonate minerals (C). **(B)** Ultrastructure of a 50 μm-thick section of the Hot Lake mat (100X magnification).

**Figure 4:**
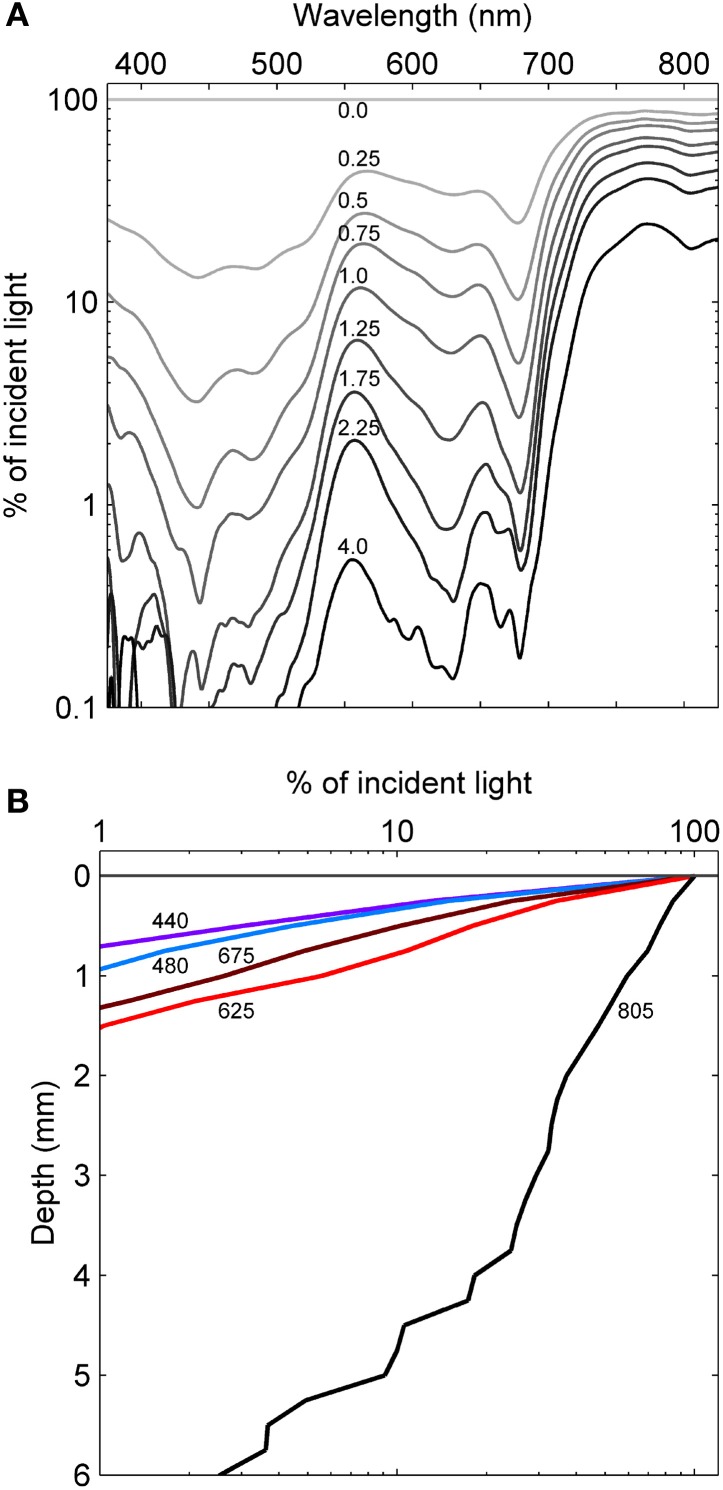
**Light penetration into the Hot Lake mat. (A)** Spectrally-resolved transmission of light through the mat as measured by fiber-optic microprobe. Numbers above the curve represent the depth the probe was inserted into the mat in millimeters. **(B)** Attenuation of wavelengths representing local maxima in absorbance. Values denote wavelength in nanometers.

The initial assembly of the mat began with stabilization of benthic sediments by a thin and gelatinous ~1 mm-thick, light-green biofilm lacking apparent lamination. As the season progressed, this biofilm matured into a coherent microbial mat characterized by a firm, rubbery texture and three to four visibly-apparent lamina (Figure 3). The dorsal surface layer of the mat was orange (Figure 3A), which microscopic examination revealed to be dominated by filamentous cyanobacteria (Figure 3B) occasionally interspersed with diatoms (data not shown). Over the seasonal cycle, the orange color of the surface layer intensified. Immediately below the orange layer was a ~1–2-mm thick, green layer dominated by filamentous cyanobacteria. The green layer was typically underlain by a pink layer composed of highly pigmented microclusters of microorganisms. Magnification (100X) also revealed a ~200–400 μm-thick brown layer sandwiched between the green and pink layers (Figure 3B). A patchy gray-black layer was occasionally observed underneath the pink layer. Inclusions of calcium and magnesium carbonates and other mineral phases were interspersed throughout the mat as observed by x-ray diffraction and electron microscopy (data not shown). Light penetration profiles measured using fiber optic microprobes revealed rapid attenuation (>99% within the first 1.0–1.5 mm, Figure 4A) of wavelengths strongly absorbed by chlorophyll *a* (with absorbance maxima of ~440 and ~675 nm) and phycocyanin (with maximum absorbance of ~625 nm). In contrast, near-infrared light (λ = 805 nm) reached the bottom of the mat, though an inflection point in the curve between 3–4 mm in depth (Figure 4B) suggested utilization by mat phototrophs. These transmission curves were generally consistent with vertical variations in pigmentation (cf. Figures 3, 4).

### Community structure of the hot lake mat around the seasonal cycle

To interrogate the spatiotemporal variability of the mat community's structure around the seasonal cycle, we collected two independent mat samples from the same location on April 21, July 7, September 1, and October 20, 2011. In each case, the mat sampled was morphologically consistent with the mature mat described above. Amplicons from the V4 hypervariable region within the 16S rRNA gene were sequenced to assay mat community structure, yielding a total of 1,470,056 assembled contigs generated from paired-end reads (exclusive of mock communities). We retained 1,207,584 quality-filtered sequences after processing. The calculated per-base error rate after completion of processing and subsampling to the size of the smallest group (14,562 reads) was ~0.029%.

**Figure 5:**
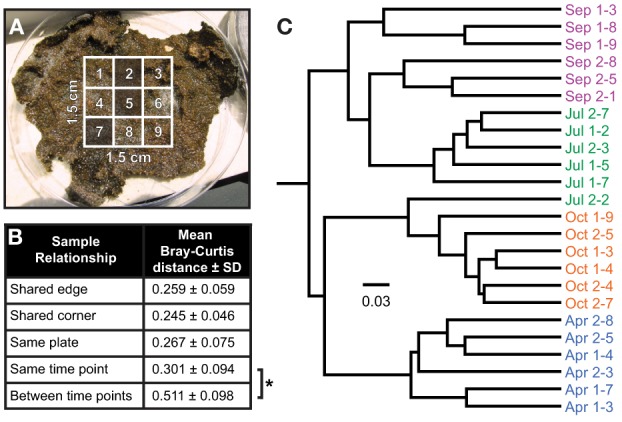
**Inter-sample variability in community structure. (A)** Random sampling strategy. A grid comprising nine subsamples, each 5 mm on a side and encompassing the entire depth of the mat (usually 3–5 mm), was cut into the center of each mat sample, three of which were selected for sequencing per plate using a random number generator. Two plates containing independent mat samples were subsampled at each time point. **(B)** Mean Bray-Curtis distance as a function of the relationship between two samples. No significant difference in mean Bray-Curtis distance, as determined by unpaired Student's *t*-test assuming unequal variance, was detected between samples that share an edge (e.g., sample 2 and 5 in panel **A**) or corner (e.g., sample 5 and 7), or non-contiguous samples from the same plate (e.g., sample 2 and 7) or on different plates collected at the same sampling time point. A significant difference was observed between samples collected at the same time point and those collected at other time points (as denoted by the asterisk, *p* < 1 × 10^−26^). **(C)** Neighbor-joining tree of Bray-Curtis β-diversity by sample.

To assess the spatial heterogeneity of the mat, three subsamples were randomly chosen from each of the two larger mat samples (a “plate”) from every time point (Figure 5A), and the distance between each of these communities was compared using the Bray-Curtis β-diversity metric (Bray and Curtis, 1957). The mean distances were then compared across time points by sample interrelationship (i.e., having a shared edge or corner, subsampled from the same plate or time point, or from different time points). No significant difference (*p* > 0.05) was observed for samples collected at the same time point, no matter their spatial relationship; however, communities were significantly more closely related to others from the same time point than those from other time points (*p* ~ 1 × 10^−27^, Figure 5B). A dendrogram of the Bray-Curtis distance matrix is represented in Figure 5C. In general, subsamples clustered strongly with others from the same time point, though the July 2-2 subsample clustered with those from October. This clustering was likely driven by a substantially smaller number of reads in July 2-2 from OTUs otherwise observed near the bottom of the mat in July (e.g., OTU 223, 229, and 231, see Figure 7) as compared with other July samples. For samples collected at the same time point, inter-plate relationships were not significantly different from intra-plate relationships with the exception of the September subsamples. In this case, the difference was again driven by a significantly reduced (*p* < 0.05) abundance of reads in subsamples derived from plate 1 associated with OTUs commonly observed near the bottom of the mat (e.g., OTU 261). These data suggest either that the mat was relatively homogeneous laterally or that the heterogeneity of community structure generally occurred at a spatial resolution much smaller than the sample size (25 mm^2^).

**Figure 6:**
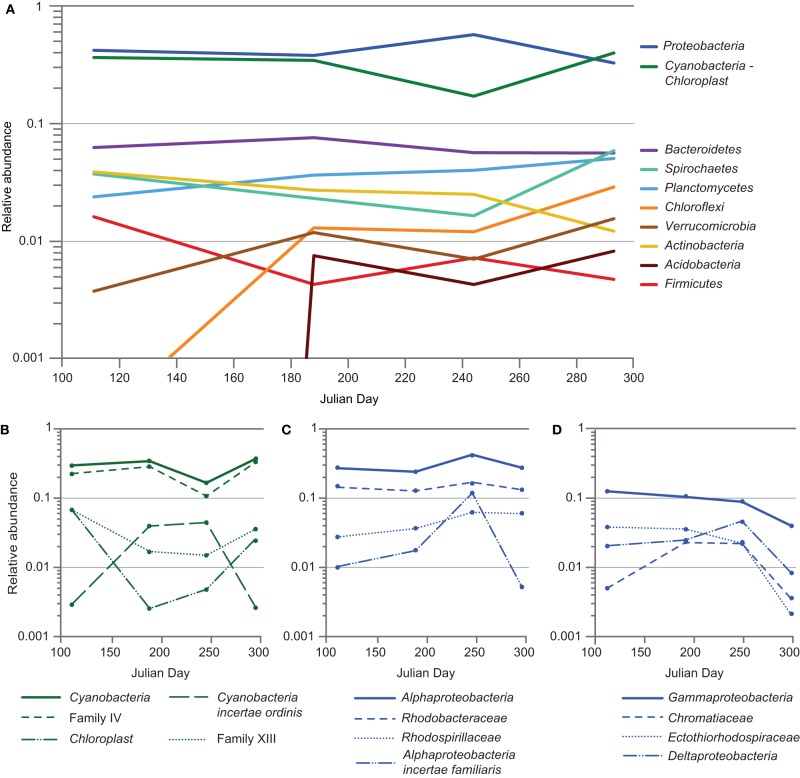
**Seasonal cycling of phylotypes within the Hot Lake mat community. (A)** Variation in major phyla of the mat community. Phyla representing > 0.5% of the reads for at least one time point were included. **(B)** Variation in classes *Cyanobacteria* and *Chloroplast*. Family IV, Family XIII, and *Cyanobacteria incertae ordinis* represent subordinate taxa of class *Cyanobacteria*. **(C)** Variation in class *Alphaproteobacteria* and subordinate families *Rhodobacteraceae*, *Rhodospirillaceae*, and *Alphaproteobacteria incertae familiaris*. **(D)** Variation in *Gammaproteobacteria* and *Deltaproteobacteria*. Families *Chromatiaceae* and *Ectothiorhodospiraceae* are subordinate families of class *Gammaproteobacteria* and, like *Deltaproteobacteria*, contain many members involved in dissimilatory sulfur cycling.

**Figure 7:**
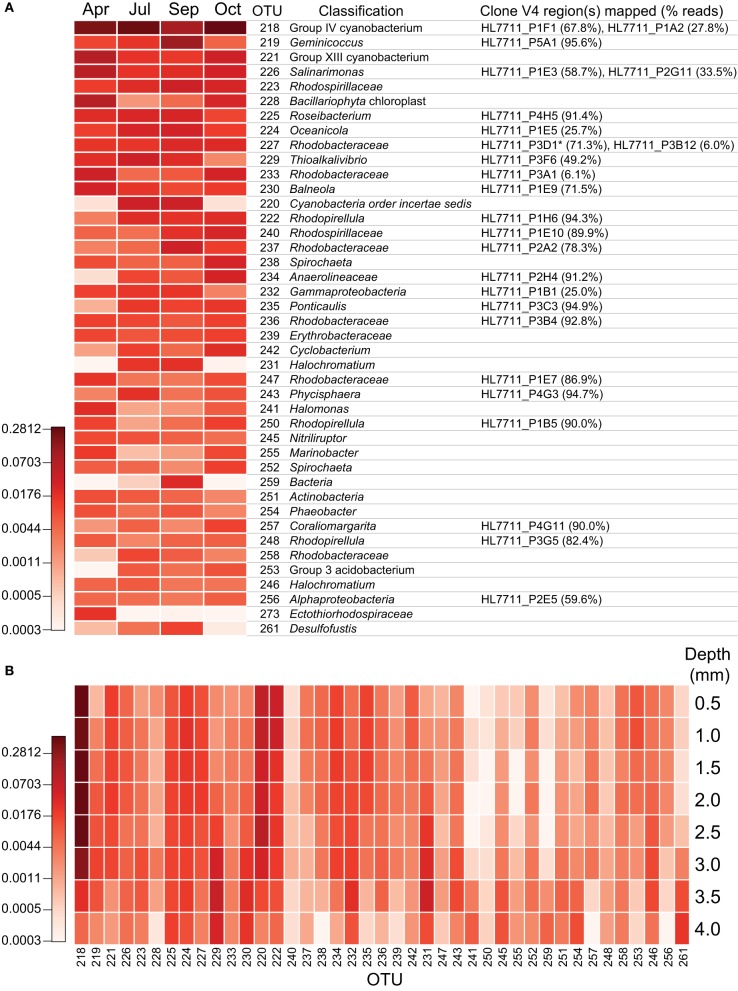
**Depth-resolved and seasonal abundance of major operational taxonomic units in the mat community.** Intensity of color depicts log_2_ transformed relative abundance data. **(A)** Seasonal cycling of major mat OTUs. After processing, reads were clustered at 97% identity using the average neighbor algorithm as implemented in mothur v. 1.29 and classified by kmer analysis using the Ribosomal Database Project training set 9 (released 3/20/2012). Each unique sequence was also mapped to the corresponding regions of the near full-length 16S sequences using the nucmer algorithm (as implemented in MUMmer 3.23). Short reads were considered to match full-length sequences if they were >99% identical across the entire amplified region. As near full-length sequences were also classified using the same protocol as the short reads, the classification with the best taxonomic resolution or bootstrap value was reported for an OTU as long as > 50% of its reads mapped to the corresponding near full-length sequence. ^*^HL7711_P3D1 shares its V4 region with HL7711_P3F7 and HL7711_P3G11. **(B)** Depth-resolved abundance of major OTUs within mat sampled on July 7, 2011 and cryosectioned. OTUs are identical to those in panel A with the exception of OTU 273, which is omitted due to a lack of reads in the depth-resolved samples. Depths reported are the maxima for each sample and represent a 0.5 mm-thick laminar section.

The mat community remained relatively stable in composition from April to October of 2011, despite an approximately tenfold increase in salinity. At the phylum level, members of *Cyanobacteria-Chloroplast* and *Proteobacteria* dominated the community throughout the seasonal cycle (Figure 6A). A statistically significant (*p* < 0.05) increase in *Proteobacteria* and concomitant decrease in *Cyanobacteria-Chloroplast* was observed in September, driven largely by a spike in sequences associated with OTU 219 and a decrease in those within OTU 218, a group IV cyanobacterium (Figures 6B, 7). OTU 219 was classified as *Geminicoccus* (Figures 6C, 7), which has not been placed into a family within *Alphaproteobacteria* (*incertae familiae*, Foesel et al., 2007). Within *Cyanobacteria*, while OTU 218 was dominant throughout 2011, the group XIII cyanobacterium OTU 221 was more abundant during periods of low irradiance. In contrast, OTU 220, classified as *Cyanobacteria incertae ordinis*, exhibited a pattern inverse to that of OTU 221. Sequences were classified using the RDP Classifier (Wang et al., 2007), which groups cyanobacteria based upon molecular similarity. Cyanobacterial sequences that did not map to near-full-length 16S sequences obtained from the clone library were compared using NCBI BLAST to all non-redundant sequences in GenBank to identify the most similar cultured representative. From that analysis, the nearest cultured neighbors of OTU 221 and 220 were found to be *Phormidium* sp. UTCC 487 (99.6% identical, Casamatta et al., 2005) and *Leptolyngbya rubra* PCB9602-6 (98% identical, A. Lopez-Cortes, unpublished data), respectively. Sequences derived from diatom chloroplasts of genus *Halamphora* and *Nitzschia* (Pillet et al., 2011), were commonly observed in spring and fall but rare during the summer (Figure 6B).

We observed the most significant (*p* < 0.05) variations in mat community composition at the phylum level between April and July and again between September and October. Between April and July, reads attributed to *Chloroflexi* (~41-fold), *Verrucomicrobia* (~3.1-fold), and *Acidobacteria* (of which no reads were observed in any of the six April samples) significantly increased, while those attributed to *Firmicutes* diminished four-fold. Between September and October, *Spirochaetes, Chloroflexi*, and *Verrucomicrobia* reads increased ~3.5-, 2.4-, and 2.2-fold, respectively, and reads attributed to *Actinobacteria* decreased slightly more than twofold. The rise in *Chloroflexi* was driven almost entirely by one OTU classified within family *Anaerolinaceae* (Figure 7A, OTU 234); very few reads attributed to family *Chloroflexaceae* were observed. We detected less than two-fold variation in relative abundance between time points for reads attributed to all other phyla accounting for ≥0.5% of reads. While the primer set employed in this study (515F-806R) is known to broadly cover archaea (Walters et al., 2011), attributed reads did not exceed 0.5% at any point. Stability in the mat community's structure throughout 2011 was also generally observed at higher taxonomic resolution (Figure 7).

Within the phylum *Proteobacteria*, reads attributed to clades affiliated with sulfur cycling strongly increased from April to July and held steady throughout the summer but had decreased precipitously by mid-autumn. *Deltaproteobacteria* and *Gammaproteobacteria* diminished approximately five- and two-fold, respectively, from September to October. The majority of the loss borne by *Gammaproteobacteria* occurred in families *Chromatiaceae* and *Ectothiorhodospiraceae*, whose members are frequently involved in sulfur cycling, diminishing approximately six- and ten-fold, respectively (Figure 6D). Much of this diminution was focused within OTUs 229 and 231, classified within the aerobic sulfide-oxidizing genus *Thioalkalivibrio* and the purple sulfur bacterium *Halochromatium* (Figure 7). Coupled with the significant concurrent reductions in phylotypes associated with sulfate reduction, as exemplified by OTU 261 (*Desulfofustis*), the data suggest that the rate of sulfur cycling may have substantially diminished between September and October.

**Figure 8:**
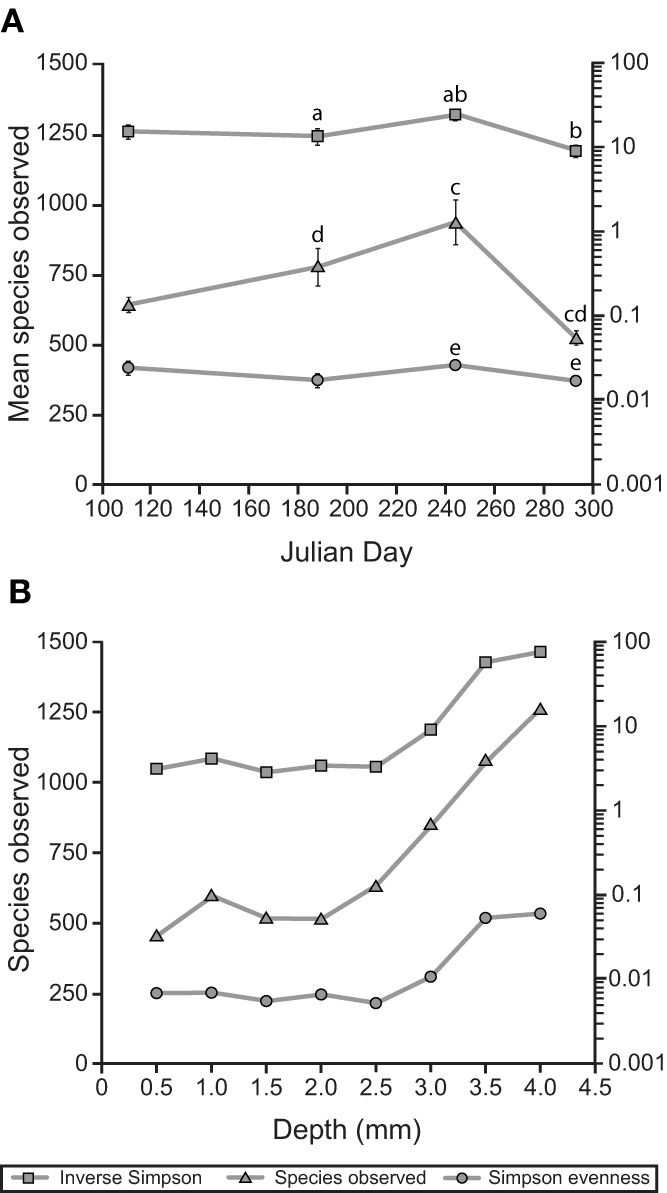
**Alpha diversity of the Hot Lake mat community. (A)** Alpha diversity, richness, and evenness around the seasonal cycle. Unitless Simpson values are plotted on the left axis. Error bars represent standard error of the mean. Statistically significant differences (*p* < 0.05) are labeled above the point with the same letter. **(B)** Depth gradient in alpha diversity, richness, and evenness. Unitless Simpson values are plotted on the left axis. Depths are reported as the maximum for each sample (i.e., 0.5 mm denotes 0–0.5 mm).

The reduction in sulfur-cycling phylotypes was part of a larger trend in reduced α-diversity in the mat community in October. After a summer season of gradually increasing trends in species observed and the inverse Simpson index, both of these metrics, as well as the Simpson evenness index, significantly declined in October (Figure 8A). In July, depth-resolved phylotype abundances revealed that members of *Cyanobacteria* (OTUs 218, 221, and 220) rapidly diminish between depths of 2.5 and 3.0 mm, where phylotypes associated with sulfide-oxidizing or anaerobic metabolisms increased sharply (for example, OTUs 229, 231, 261, Figure 7B). These data suggest the presence of a sharp chemocline at this position in the mat, which is consistent with the light profile in suggesting a termination of oxygenic photosynthesis below this depth (Figure 4). The bottom of the mat exhibited significantly increased α-diversity by all metrics (species observed, inverse Simpson, and Simpson evenness indices, Figure 8B), and OTUs observed at or near the bottom of the mat in July were largely absent in October (Figure 7B). These data suggest that mat community disassembly, defined as the combined processes of biomass turnover and dispersion of cells as mat exopolymer is degraded, is associated with the loss of the phylotypes that inhabit the bottom of the mat.

**Figure 9:**
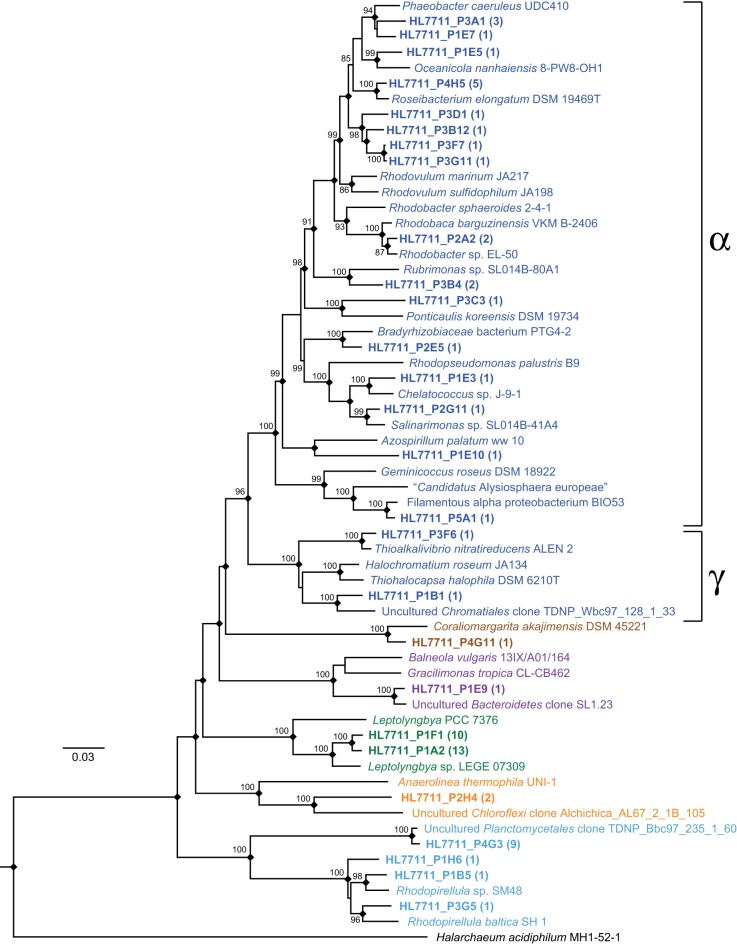
**Phylogenetic reconstruction of near full-length 16S sequences from the Hot Lake mat representing major OTUs.** Clones were generated from mat sampled on July 7, 2011 and are in bold. Clusters of sequences with >99% identity are represented by a single sequence; the number of sequences represented by each is noted parenthetically. While a neighbor-joining tree is depicted above, nodes duplicated using a maximum-likelihood algorithm employing the general time-reversible model are notated with a diamond. Values near nodes represent neighbor-joining bootstrap values greater than 80. Terminal node colors denote phyla according to the same scheme used in Figure 6A. Classes *Alphaproteobacteria* and *Gammaproteobacteria* are enclosed in brackets.

In order to improve the phylogenetic resolution afforded by the short reads, we mapped them to the analogous region of the near-full-length clone sequences. The result of mapping reads from the most abundant OTUs to the clones is detailed in Figure 7 (≥99% identity). In most cases, the OTUs were dominated by a single sequence, and thus, mapped to a single clone (e.g., OTUs 219, 225, 222). In other cases, the OTUs contained sequences mapping to several clones (e.g., OTUs 218, 226, 227) or a single clone recruited only a small fraction of the reads in an OTU (e.g., OTUs 224, 233, 232). In many cases, mapping to the clones allowed the OTUs to be classified phylogenetically at much greater resolution. A neighbor-joining phylogeny of clones mapped by the reads from the most abundant OTUs and their nearest neighbors is represented in Figure 9. In some cases, this mapping made otherwise inscrutable relationships apparent; for example, clones HL7711_P1F1 and HL7711_P1A2 are 98.2% identical, having only two regions of difference, one of which is covered by the Itags. Mapping to the longer clones revealed that the ratio of reads mapping to HL7711_P1F1 vs. HL7711_P1A2 is ~2:1 in all samples examined (95% confidence interval 1.76–2.98), suggesting that they are from divergent 16S rRNA genes on the same cyanobacterial chromosome. In contrast, OTU 229 was dominated by two sequences sharing 98.8% identity and of roughly equal abundance, one for which a matching clone (HL7711_P3F6) was available, and one that did not match a clone at ≥99% identity. The ratio between these sequences was inconsistent (averaging 3.00 ± 6.53, standard deviation) and the abundance of each was decoupled in space (depth-resolved abundance in July) and time. Reads from OTU 229 mapping to HL7711_P3F6 were more abundant in April and July, while the other sequence was more frequently observed in September (data not shown), implying that these sequences represent different organisms. Collectively, these data suggest that the depth of coverage afforded by Itag sequencing allows increased dissection of the internal structure of an OTU's composite sequences. In some cases, this provides more detailed insight into the variation of individual species or ecotypes that may be combined into single OTUs by clustering algorithms.

### Inference of ecological drivers of community structure by phylogenetic turnover

We examined variation at the OTU level in light of sample metadata using phylogenetic turnover analysis to infer the ecological parameter(s) most responsible for driving variations in community structure. One set of analyses used Bray-Curtis, which quantifies turnover in the relative abundance of OTUs. A second set of analyses used βNTI, which measures the deviation between observed and expected phylogenetic turnover, reported as βMNTD. Phylogenetic turnover (i.e., βMNTD) quantifies the difference in phylogenetic composition between a given pair of communities. For example, βMNTD will be small if OTUs within one community are closely related to the OTUs in a second community. Likewise, βMNTD will be large when OTUs within one community are distantly related to OTUs in a second community. Randomizations provide an expected level of βMNTD under the assumption that the observed magnitude of Bray-Curtis is due to stochastic changes in OTU abundances. The value of βNTI is the difference between observed and expected βMNTD. In turn, increasingly large βNTI values indicate an increasing influence of deterministic processes that select upon environmentally-determined fitness to cause differences in OTU relative abundances.

All Mantel tests relating Bray-Curtis to environmental variables were significant (p « 0.05 for all), while βNTI was significantly related only to the standard deviation across one week of irradiance. In addition, while Bray-Curtis increased with environmental distance for all variables, βNTI decreased with increasing environmental distance for over half of the environmental variables. The use of phylogenetic turnover to make ecological inferences was supported by significant phylogenetic signal, but only within relatively short phylogenetic distance classes, as has been previously observed (Andersson et al., 2009; Stegen et al., 2012, 2013; Wang et al., 2013). Significant phylogenetic signal across short phylogenetic distances specifically supports the use of βMNTD and βNTI, as these metrics quantify phylogenetic turnover among nearest phylogenetic neighbors; our analyses indicate that the assumption of phylogenetic signal is most likely supported across short phylogenetic distances (see also Stegen et al., 2012, 2013).

Our interpretations of the Mantel test results are necessarily conservative because communities were only sampled across four points in time such that there are only four independent estimates of environmental conditions. There are, nonetheless, patterns that point toward specific environmental factors that drive variation in community composition. In particular, Mantel tests using Bray-Curtis or βNTI both suggest that temporal variation in light availability most strongly influenced the community composition of the Hot Lake mat among measured environmental variables. Two variables in the Bray-Curtis analysis had noticeably higher correlation coefficients relative to all other variables, and both were related to variation in light. Only one environmental variable was significantly (albeit very weakly) related to βNTI, and this variable was again related to variation in light. Taken together, these data suggest that the structure of the Hot Lake mat community was more strongly influenced by the dynamics of photic energy than by changes in either water temperature or salinity.

## Discussion

Within Hot Lake, a single mat community is annually exposed to nearly 10-fold changes in the concentrations of Mg^2+^, SO^2−^_4_, and other dissolved ions. Although the role of increasing salinity in restricting microbial diversity and activity within mat communities has been well-established (e.g., Pinckney et al., 1995; Benlloch et al., 2002; Sorensen et al., 2005; Severin et al., 2012), relatively few studies have investigated the impact of salinity on the structure of mat communities exposed to naturally occurring salt concentration dynamics. Previous studies examining the impacts of variable salinity on community structure have frequently focused upon the sequential pools of solar salterns (reviewed in Oren, 2009) where salinity is relatively well-controlled, and high-evaporation intertidal mats such as those near Abu Dhabi (Abed et al., 2007). In the case of solar saltern systems, the mats of sequential concentrating pools are largely end-members (vis-à-vis salinity) and must be treated as discrete neighboring communities. In the case of tide pool salinity cycling, the mat community is repeatedly exposed to maximal salinity for relatively short durations. In contrast, like other microbial mats exposed to significant natural variation in salinity (e.g., Yannarell et al., 2006; Desnues et al., 2007; Yannarell and Paerl, 2007), the Hot Lake microbial mat community must annually adapt to salinity conditions ranging from brackish to extremely hypersaline.

Given that previous work (Jungblut et al., 2005; Rothrock and Garcia-Pichel, 2005; Abed et al., 2007) has demonstrated a salinity limitation on species diversity in cyanobacterial mats, we sought to determine whether the seasonally-increasing salinity of Hot Lake would promote a succession of cyanobacteria with increasing epsotolerance (Nübel et al., 2000, cf. Table 1). Our data suggest, rather, that a single cyanobacterium (*Leptolyngbya*) is dominant throughout the seasonal cycle. While other, less abundant cyanobacteria and diatom chloroplasts exhibit significant seasonal variation (Figure 7A, OTUs 221, 228, and 220), their patterns of variation correlate more strongly with irradiance and/or temperature than with salinity. In general, the cyanobacterial species occupying the Hot Lake mat appear to be similar to those in communities observed in high-latitude and polar mats (Jungblut et al., 2005, 2009; Fernandez-Carazo et al., 2011; Kleinteich et al., 2012; Martineau et al., 2013) with dominant populations of *Phormidium* (e.g, OTU 221) and *Leptolyngbya* (OTUs 218 and 220) species. Of note is the absence of the nearly-ubiquitous mat-building cyanobacterium *Coleofasciculus chthonoplastes* (Guerrero and De Wit, 1992; Jonkers et al., 2003). Although Hot Lake cycles through salinities well known to be permissive for *Coleofasciculus*, there was no microscopic or molecular evidence for the presence of this cyanobacterium. The cyanobacteria detected in our study are consistent with the microscopic observations of Anderson and collaborators, suggesting that the same cyanobacteria may have anchored the mat community for the past 55 years despite significant changes in lake level over that time (Anderson, 1958).

In general, the non-cyanobacterial fraction of the mat community also exhibits relative stability over the course of the seasonal cycle at fine taxonomic resolution. One notable exception is the loss of OTUs likely to be involved in sulfur cycling (i.e., *Deltaproteobacteria*, and, within *Gammaproteobacteria*, families *Ectothiorhodospiraceae* and *Chromatiaceae*, Figure 6D) and other OTUs populating the lower regions of the mat late in the seasonal cycle. This loss occurred during a period of little change in the salinity of overlying water and contributed to reductions in species observed, Simpson evenness, and inverse Simpson metrics between late summer and late fall (Figure 7A). As the bottom ~1 mm of the mat is considerably more diverse than the overlying cyanobacterially-dominated laminae, elimination of niches near the mat-sediment interface has an amplified impact upon the overall α-diversity of the mat community.

Mantel tests relating β NTI to environmental variables did not support a strong linkage between community composition and salt concentration. One possible explanation is that salinity increases rapidly enough in Hot Lake throughout the seasonal cycle that organisms with broad epsotolerance are positively selected. The cyclical nature of this selective pressure may have generated a regional pool of broadly epsotolerant potential mat members that numerically dominate and, through priority effects, exclude organisms with higher fitness across a narrower range of salt concentrations. If true, this predicts that the competitiveness of mat taxa should not vary systematically across the range of salt concentrations endemic to Hot Lake. Our analyses also suggest that the observed reductions in diversity between September 1 and October 20, 2011 are part of a larger correlation between increased variation in irradiance and decreased OTU richness. Classic coexistence theory (e.g., Chesson, 2000) predicts that temporally variable environments should promote coexistence and, in turn, increase OTU richness. We related OTU richness to the standard deviation in irradiance, which exhibited a strong negative relationship. This pattern leads us to hypothesize that community composition shifts through time, at least in part due to the exclusion of some taxa by ecological selection imposed by temporal variation in light. This selection might be mediated by the availability of metabolizable organic carbon at low or variable irradiances, as some evidence suggests cyanobacteria exude increased amounts of low molecular weight organic carbon molecules under elevated irradiances (Zlotnik and Dubinsky, 1989; Lee and Rhee, 1999).

Our ability to infer the ecological drivers of the mat community structure is limited by the temporal resolution in sampling with respect to changes in environmental parameters. For example, we measured salt concentrations of ~28, 118, 251, and 252 mg/L, such that, effectively, there were only three distinct levels evaluated in our analyses. Furthermore, because βNTI is sensitive to relative abundance, it is also possible that the high abundance and relative stability of the cyanobacterial and alphaproteobacterial compartment of the Hot Lake mat obscure the effect of important salinity-mediated changes in less abundant but functionally significant OTUs (e.g, in *Gammaproteobacteria* and *Deltaproteobacteria*). These changes in taxa associated with sulfur cycling may be harbingers or drivers of overwinter mat community disassembly processes. In order to more robustly evaluate the hypothesis that variation in light, rather than salinity, is structuring the Hot Lake microbial mat community, additional measurements of the mat community structure and local physicochemical properties taken at greater temporal resolution will be required.

Taken together, our observations suggest two hypotheses for the loss of the Hot Lake mat's relatively rich and even understructure. The first is that the seasonal reduction and increased variability of irradiance (Figure 2B), which result in diminished photosynthesis, reduce the amount of energy and reduced carbon available for the maintenance of heterotroph biomass. This effect might be felt both in the growth rate of primary producer biomass that can be recycled and the amount of low molecular weight photosynthate reaching the bottom regions of the mat, where dissimilatory sulfate reduction and fermentation are likely to be main metabolic strategies. Primary productivity is known to diminish with increasing salinity (Pinckney et al., 1995); this may further limit the availability of reduced carbon and nitrogen species to heterotrophs and favor the net consumption of extracellular polymers (Braissant et al., 2009).

A second hypothesis is that increasing salinities, which require energetically expensive osmotic regulation, eventually exclude species with low energy-yielding metabolisms. Although the extreme sulfate concentrations of Hot Lake make dissimilatory sulfate reduction more energetically favorable than in an equisaline NaCl environment, sulfate reduction appears to exhibit a global salinity maximum for metabolic viability (Oren, 2011). If sulfate reduction (e.g., by *Deltaproteobacteria*) is negatively impacted by elevated salinities, reductions in sulfide oxidizers within *Ectothiorhodospiraceae* and *Chromatiaceae* are likely to closely follow. As the turnover rate of organisms within the mat is unknown, the phylogenetic signals may lag significantly behind decreases in metabolic activities. This may account for the observed change in relative abundance of these phylotypes over a period of stable salinity (September 1 to October, 20 2011). Quantifying the reaction rates of photosynthesis, sulfide oxidation, and sulfate reduction with respect to the relative abundances of associated phylotypes throughout the seasonal cycle will help to discern which of these hypotheses best explain our observations.

We expect that elucidation of the major ecological variables governing the Hot Lake microbial mat community will shed light on the environmental parameters driving its seasonal assembly and disassembly. Seasonal disassembly of a microbial mat is by no means unique to Hot Lake. Mats inhabiting diverse habitats, such as the salt marshes of Sippewissett and the North Sea barrier island beaches of Mellum, are frequently destroyed over the winter (Stal et al., 1985; Franks and Stolz, 2009), and tropical mats are known to be destroyed by hurricanes (Yannarell et al., 2007). The action of wind, waves, and tides are believed to be the primary means for the physical destruction of such microbial mats. Renaut considered potential mechanisms of microbial mat destruction in saline lakes and playas of the Cariboo Plateau, British Columbia, with an eye toward their potential for preservation within the geological record (Renaut, 1993). Of the seven mechanisms he proposed for destruction of Cariboo Plateau mats, diagenetic decomposition, which we deem equivalent to mat disassembly, seems to be the most likely explanation for the Hot Lake mat's overwinter disappearance from supralittoral and benthic surfaces.

Our observations suggest the hypothesis that the mat community assembles during periods in which solar energy is abundant and the rate of photosynthesis is correspondingly high. Rates of photosynthesis that exceed consumption may drive the accumulation of carbon-rich extracellular polymers that compose the mat's matrix and provide opportunities for the recruitment of new mat members with diverse metabolic capacities and narrow physicochemical tolerances. Conversely, when the rate of heterotrophic degradation of these polymers exceeds their rate of synthesis, the mat community may begin to disassemble as the matrix is consumed and niches are lost. Hot Lake, therefore, presents a unique opportunity to study the recruitment of metabolic function to an assembling community and the corresponding loss of function as the community disassembles (Johnson et al., 2012). Metagenome-enabled study of the Hot Lake mat community may uncover the interspecies metabolic interactions responsible for mat formation and stability and aid in the elucidation of design principles for microbial community assembly.

### Conflict of interest statement

The authors declare that the research was conducted in the absence of any commercial or financial relationships that could be construed as a potential conflict of interest.
