# Supplementary material for: The epsomitic phototrophic microbial mat of Hot Lake, Washington: community structural responses to seasonal cycling
Source: Front Microbiol. 2013 Nov 13;4:323. doi: 10.3389/fmicb.2013.00323 (PMC3826063; doi:10.3389/fmicb.2013.00323)
Supplement: Supplementary file 1 [file Presentation1.PDF]

Supplemental Table 1: Accession numbers referenced in this study

## Near neighbors

| Sequence | GenBank Accession Number |
|---|---|
| "*Candidatus* Alysiosphaera europeae" | AY428766.1 |
| *Anaerolinea thermophila* UNI-1 | NR_074383.1 |
| *Azospirillum palatum* ww 10 | EU747318.1 |
| *Balneola vulgaris* 13IX/A01/164 | AY576749.1 |
| *Bradyrhizobiaceae* bacterium PTG4-2 | EU603455.1 |
| *Chelatococcus* sp. J-9.1 | FR774565.1 |
| *Coraliomargarita akajimensis* | AB266750.1 |
| Filamentous alpha proteobacterium BIO53 | AY590699.1 |
| *Geminicoccus roseus* DSM 18922 | AM403172.1 |
| *Gracilimonas tropica* CL-CB462 | EF988655.1 |
| *Halarchaeum acidiphilum* MH1-52-1 | AB371717.2 |
| *Oceanicola nanhaiensis* 8-PW8-OH1 | HQ425690.2 |
| *Phaeobacter caeruleus* UDC410 | HM031996.1 |
| *Ponticaulis koreensis* DSM 19734 | NR_044608.1 |
| *Rhodobaca barguzinensis* VKM B-2406 | EF554833.1 |
| *Rhodobacter* sp. EL-50 | AJ605746.2 |
| *Rhodobacter sphaeroides* 2.4.1 | NR_074171.1 |
| *Rhodopirellula baltica* SH 1 | NR_043384.1 |
| *Rhodopirellula* sp. SM48 | FJ624354.1 |
| *Rhodopseudomonas palustris* B9 | KC162163.1 |
| *Rhodovulum marinum* JA217 | AM696301.1 |
| *Rhodovulum sulfidophilum* JA198 | AM696694.1 |
| *Roseibacterium elongatum* | FN667962.1 |
| *Rubrimonas* sp. SL014B-80A1 | GU125652.3 |
| *Salinarimonas* sp. SL014B-41A4 | GU125653.2 |
| *Thioalkalivibrio nitratireducens* ALEN 2 | AY079010.1 |
| *Thiohalocapsa halophila* DSM 6210T | AM903380.1 |
| Uncultured *Bacteroidetes* bacterium clone SL1.23 | JX240555.1 |
| Uncultured *Chloroflexi* bacterium clone Alchichica_AL67_2_1B_105 | JN825481.1 |
| Uncultured *Chromatiales* bacterium clone TDNP_Wbc97_128_1_33 | FJ517010.1 |
| Uncultured *Planctomycetales* bacterium clone Alchichica_AL52_2_1B_172 | JN825619.1 |
| Uncultured Planctomycetales bacterium clone TDNP_Bbc97_235_1_60 | FJ516780.1 |

## Hot Lake clones

| Sequence | GenBank Accession Number |
|---|---|
| HL7711_P1B1 | KC896650.1 |
| HL7711_P1F1 | KC896651.1 |
| HL7711_P1A2 | KC896652.1 |
| HL7711_P1B2 | KC896653.1 |
| HL7711_P1G2 | KC896654.1 |
| HL7711_P1C3 | KC896655.1 |
| HL7711_P1E3 | KC896656.1 |
| HL7711_P1B5 | KC896657.1 |
| HL7711_P1E5 | KC896658.1 |
| HL7711_P1H6 | KC896659.1 |
| HL7711_P1E7 | KC896660.1 |
| HL7711_P1E9 | KC896661.1 |
| HL7711_P1E10 | KC896662.1 |
| HL7711_P2A2 | KC896663.1 |
| HL7711_P2B2 | KC896664.1 |
| HL7711_P2C2 | KC896665.1 |
| HL7711_P2E2 | KC896666.1 |
| HL7711_P2F8 | KC896667.1 |
| HL7711_P2H4 | KC896668.1 |
| HL7711_P2H5 | KC896669.1 |
| HL7711_P2G11 | KC896670.1 |
| HL7711_P3A1 | KC896671.1 |
| HL7711_P3D1 | KC896672.1 |
| HL7711_P3C3 | KC896673.1 |
| HL7711_P3B4 | KC896674.1 |
| HL7711_P3G5 | KC896675.1 |
| HL7711_P3F6 | KC896676.1 |
| HL7711_P3F7 | KC896677.1 |
| HL7711_P3C8 | KC896678.1 |
| HL7711_P3G11 | KC896679.1 |
| HL7711_P3B12 | KC896680.1 |
| HL7711_P4H1 | KC896681.1 |
| HL7711_P4G3 | KC896682.1 |
| HL7711_P4E4 | KC896683.1 |
| HL7711_P4B6 | KC896684.1 |
| HL7711_P4A7 | KC896685.1 |
| HL7711_P4F7 | KC896686.1 |
| HL7711_P4G11 | KC896687.1 |
| HL7711_P5A1 | KC896688.1 |
